# Managing Local Bleeding in Dentoalveolar Surgery: Practical Techniques and Best Practices: A Narrative Review

**DOI:** 10.31661/gmj.v13iSP1.3686

**Published:** 2024-12-29

**Authors:** Fatemeh Rahmani, Dorsa Nikeghbal, Sayeh Zarehgar, Meysam Mohammadikhah, Seyed Sasan Aryanezhad, Reza Mahmoudi Anzabi, Mehran Zareanshahraki, Ali Goodarzi

**Affiliations:** ^1^ Oral and Maxiofacial Surgery Department, Shiraz Dental School, Shiraz University of Medical Science, Shiraz , Iran; ^2^ Student Research Committee, School of Dentistry, Shiraz University of Medical Sciences, Shiraz, Iran; ^3^ Department of Prosthodontics , School of Dentistry, Unciano Colleges and General Hospital , Manila, Philipines; ^4^ Department of Oral and Maxillofacial Surgery, School of Dentistry, Alborz University of Medical Sciences, Karaj, Alborz, Iran; ^5^ Oral and Maxillofacial Radiologist , Private Practice, Tehran , Iran; ^6^ Department of Orthodontics, Faculty of Dentistry, Tabriz University of Medical Sciences, Tabriz, Iran; ^7^ UCLA School of Dentistry Preceptorship Surgical Implant Dentistry, Los Angeles, State, California, United States of America; ^8^ Department of Periodontics, School of Dentistry, Shiraz University of Medical Sciences, Shiraz, Iran

**Keywords:** Dentoalveolar Surgery, Hemostasis, Surgical Hemorrhage Control Anticoagulants, Hemostatic Agents

## Abstract

Background: Effective management of local bleeding is essential for safe and successful dentoalveolar surgery, particularly among patients with bleeding disorders, those on anticoagulant therapy, or individuals with systemic conditions affecting hemostasis. This narrative review explores practical and advanced approaches for controlling bleeding in dentoalveolar procedures, with a focus on patient-centered strategies that minimize risk while accommodating complex clinical needs. Materials and Methods: narriative review. Results: Beginning with an overview of the pathophysiology of bleeding in the oral cavity, the review examines conventional methods such as mechanical compression, sutures, and the use of local anesthetics with vasoconstrictors. Further, it evaluates the application of pharmacological agents, including topical hemostatic products like gelatin sponges, oxidized cellulose, and tranexamic acid, which have shown efficacy in high-risk patients without disrupting systemic anticoagulation therapy. For special populations, including those with congenital coagulopathies and liver disease, tailored approaches are reviewed to address unique bleeding challenges. Additionally, innovative hemostatic materials and laser-assisted techniques are discussed as emerging options that promise enhanced safety and effectiveness in complex cases. Conclusion: By synthesizing current knowledge on bleeding control methods, this review provides clinicians with practical guidance for optimizing hemostasis in dentoalveolar surgery. The insights and recommendations presented aim to improve patient outcomes, reduce perioperative complications, and support a balanced approach to local and systemic hemostatic management in diverse patient groups.

## Introduction

Bleeding risks in dentoalveolar surgery are a critical consideration, particularly for patients on anticoagulant therapy or with conditions affecting hemostasis. Research highlights the need for careful perioperative planning and interdisciplinary collaboration to mitigate these risks effectively. For instance, studies show that while bleeding is more common in patients on uninterrupted anticoagulation therapy, it is often manageable with structured protocols and thorough patient education [1]. Similarly, patients undergoing perioperative heparin bridging face a heightened bleeding risk compared to those on continuous vitamin K antagonists, emphasizing the need for a balanced approach to anticoagulation management [1].

In patients with liver cirrhosis, where coagulation is impaired, local hemostatic measures have proven effective, maintaining a low bleeding risk during dental procedures [2]. Similarly, patients with immune thrombocytopenia or those on direct oral anticoagulants benefit from individualized treatment plans, showing minimal bleeding when local measures are employed [3].

For patients undergoing dental implant or extraction procedures, recent studies suggest that managing bleeding risks effectively can allow surgeries to proceed without altering antithrombotic regimens. For example, implant procedures in patients on anticoagulant therapy generally maintain a low bleeding risk with proper hemostatic support [4], though the use of platelet-rich fibrin (PRF) was found ineffective in further reducing postoperative bleeding [5].

A systematic review similarly highlighted the safety of implant surgery in anticoagulated patients, reinforcing the utility of local hemostatic measures [6].

Furthermore, a study on dental extractions in patients on antithrombotic medication found a low risk of postoperative bleeding when established American College of Chest Physicians guideline were followed, underscoring the importance of adherence to clinical protocols [7].

Effective control of local bleeding is essential in dentoalveolar surgery, especially for patients with bleeding disorders or those on anticoagulant therapy. Research shows that local hemostatic measures, such as tranexamic acid mouthwash and absorbable sponges, are effective in managing postoperative bleeding even in patients who continue anticoagulant medications during surgery. This approach allows for the safe continuation of necessary medications without heightening the risk of thromboembolic events [1].

For instance, studies comparing the use of continuous oral anticoagulation versus heparin bridging therapy found that simple local agents like collagen sponges adequately controlled bleeding, further emphasizing that anticoagulant interruption may not be necessary in routine procedures [8]. For high-risk patient groups, including individuals with congenital bleeding disorders or advanced liver disease, effective local bleeding control methods are similarly essential. Studies demonstrate that using localized techniques such as fibrin glue and tranexamic acid significantly reduces bleeding complications in these populations without systemic complications [9].

Additionally, in patients with liver failure who may face complex surgical needs, locally applied measures have been effective in managing bleeding and avoiding complications [10].

These findings underscore the practicality and safety of local hemostatic strategies in minimizing bleeding risks in dentoalveolar procedures across diverse patient groups.

A narrative review on this field is essential to provide a intensive understanding of the diverse techniques and strategies used to manage bleeding across different patient populations, including those with anticoagulation therapy, liver disease, and inherited bleeding disorders.

Unlike systematic reviews, a narrative approach allows for a broader discussion of both established and emerging hemostatic methods, offering insights into clinical adaptations that enhance patient safety without interrupting necessary systemic treatments. The aim of this review is to consolidate current knowledge, evaluate the efficacy of various local hemostatic agents, and present practical guidance tailored to the needs of complex patient groups, ultimately supporting improved outcomes in dentoalveolar surgery.

## Pathophysiology of Bleeding in Dentoalveolar Surgery

Vascular Anatomy and Bleeding Sources in the Oral Cavity

The oral cavity’s vascular anatomy presents numerous potential sources of bleeding due to its complex network of arteries that supply the tissues and bones involved in dentoalveolar procedures. The primary vessels at risk include branches from the facial, lingual, and maxillary arteries, which supply critical areas such as the mucosa, gingiva, and alveolar bone. In particular, the submental and sublingual arteries supply the floor of the mouth and can pose a significant bleeding risk during implant placements and other invasive procedures. The dental pulp cavity receives its blood supply from the inferior alveolar artery, which is one of the six mucosal territories of the intraoral cavity. The path of these arteries varies among individuals, with some cases showing close proximity to surgical sites, leading to increased risks of accidental laceration and hemorrhage [11].

A study confirmed that anatomical variations in the vascular supply of the oral cavity can lead to significant bleeding complications, particularly in high-risk areas like the molar region. For instance, bleeding risks are notably elevated in this area, especially during tooth extractions or other procedures that involve substantial manipulation of the gingival or alveolar tissues. This risk is further heightened in patients with congenital clotting disorders or those on anticoagulant therapy, emphasizing the need for individualized anatomical assessment prior to surgery [12].

The lower third molar area has been identified as a particularly vulnerable site, as its surrounding tissues are richly supplied by branches of the inferior alveolar artery. Studies report that this region, along with the maxillary regions in periodontal surgeries, presents an elevated risk of hemorrhage due to the dense vascular network and high rate of local trauma [13].

The dense network of small capillaries and venules in the gingival and mucosal tissues also contributes to bleeding during minor surgeries, which can complicate postoperative healing if not managed with careful hemostatic measures [14]. Additionally, a study on the use of local hemostatic agents in patients with compromised hemostasis highlights the importance of preoperative knowledge of oral vascular anatomy. By understanding these bleeding sources, clinicians can apply appropriate localized measures, such as HemCon dressings or collagen sponges, to control bleeding effectively during surgery, particularly in areas where the vascular network is dense [15]. In summary, the intricate vascular anatomy of the oral cavity, with particular bleeding risks from the sublingual, submental, and inferior alveolar arteries, demands careful consideration during surgical planning and execution. Variations in this vascular network necessitate a tailored approach to minimize intraoperative and postoperative bleeding risks, especially in high-risk areas and for patients with coagulopathies or on anticoagulation therapy.

## Mechanisms of Hemostasis Relevant to Oral Surgery

Hemostasis in oral surgery involves a series of intricate mechanisms that stop bleeding through vascular constriction, platelet activation, and coagulation cascades. These processes work in tandem to control blood loss during invasive dental procedures. Initial vasoconstriction limits blood flow at the injury site, allowing platelets to aggregate and form a temporary plug. Platelet function is crucial in this phase, especially in response to subendothelial collagen exposure, which initiates adhesion and subsequent clot formation [16]. In patients undergoing oral surgery, medications like anticoagulants and antiplatelet agents can impair this process by affecting platelet function or the coagulation pathway. For instance, a study comparing bleeding risks in patients on platelet-altering medications versus controls found no difference in blood loss in case of minor procedures without stoping platelet-altering medications [17].

Antifibrinolytic agents like tranexamic acid and epsilon aminocaproic acid are often employed in oral surgery to stabilize the fibrin clot by inhibiting fibrinolysis, preventing excessive bleeding, especially in patients with clotting disorders such as hemophilia and von Willebrand disease [18]. A study examining tranexamic acid in anticoagulated patients reported significantly reduced postoperative bleeding, demonstrating the agent’s efficacy in managing hemostasis in patients on anticoagulation therapy [19].

The coagulation cascade is also fundamental in achieving effective hemostasis during oral surgery. This process involves a series of reactions that activate thrombin, which converts fibrinogen into fibrin, creating a stable blood clot. In patients with congenital bleeding disorders, managing these mechanisms becomes challenging. For example, von Willebrand factor is essential for the initial adhesion of platelets to the injured vessel wall and for the stabilization of factor VIII [20]. Local hemostatic agents such as gelatin sponges, collagen, and fibrin sealants play a complementary role in enhancing hemostasis by providing a matrix for clot formation. These agents are particularly effective in patients with altered hemostasis, such as those on long-term anticoagulation therapy. Studies confirm that these agents help maintain localized control of bleeding without requiring systemic therapy adjustments [21][22].

In conclusion, the mechanisms of hemostasis in oral surgery rely on an orchestrated balance between vascular responses, platelet activity, and the coagulation cascade. For patients with compromised hemostatic function, the use of antifibrinolytics, local hemostatic agents, and preoperative planning tailored to their coagulation status is essential to achieving safe and effective blood loss control during surgery.

## Preoperative Assessment and Risk Factors

Preoperative assessment and identification of risk factors are essential for managing bleeding in dentoalveolar surgery, especially in patients with systemic conditions or those on medications that affect hemostasis. A comprehensive preoperative evaluation begins with a detailed history of the patient’s bleeding tendencies, family history, and medication use. Routine assessment, including coagulation screening, can help identify high-risk patients, as shown in studies indicating that history alone, though valuable, may need to be supplemented with targeted coagulation tests to catch asymptomatic bleeding disorders [23][24]. Systemic conditions such as liver disease, coagulation disorders, and the need for anticoagulation therapy significantly increase the risk of bleeding. For example, a study on liver transplant candidates undergoing extractions showed that, despite comprehensive preparation, there was still a notable risk of postoperative bleeding, underscoring the need for careful planning and prophylactic measures [25]. The use of anticoagulant and antiplatelet medications also complicates bleeding control in oral surgery. Studies indicate that while many anticoagulated patients can safely undergo extractions without modifying their medication regimen, individual risk assessment is vital to manage bleeding effectively [26].

Preoperative planning should involve multidisciplinary collaboration, especially for patients with complex medical histories. In high-risk cases, such as patients with hemophilia or severe liver disease, integrating hemostatic agents like tranexamic acid and local measures can reduce bleeding risk, though careful monitoring remains essential [27].

Additionally, guidelines by the Italian Society for Thrombosis and Hemostasis advocate for a structured approach to preoperative bleeding assessment, advising individualized testing and the judicious use of laboratory tests to reduce preventable bleeding complications [28].

Patient preparation may also involve lifestyle modifications, such as encouraging smoking cessation and addressing alcohol use, which are known to influence perioperative outcomes. Obesity, poor nutritional status, and low exercise tolerance are additional factors linked to increased surgical risks, highlighting the importance of a comprehensive assessment that accounts for lifestyle influences on hemostasis [29].

## Conventional Techniques for Bleeding Control

Conventional mechanical techniques, such as gauze packing, sutures, and pressure application, play a central role in achieving hemostasis during dentoalveolar surgeries. Studies have demonstrated that direct manual pressure with gauze is highly effective for primary hemorrhage control, particularly when multiple layers of gauze and a two-handed pressure application are used, allowing for increased compression over the bleeding site [30]. Enhanced gauze options, such as HemCon Dental Dressing (Tricol Biomedicl INC, made in USA), have shown that hemostatic-embedded gauze can reduce bleeding time significantly and improve healing in patients on anticoagulants compared to traditional pressure-only gauze [31].

Similarly, elastic adhesive dressings applied over gauze are practical and maintain hemostasis in challenging areas, providing reliable compression without compromising blood flow [32].

In addition to gauze-based methods, sutures are widely used to control bleeding by securing clots and stabilizing tissues, especially useful in closing small vessels and managing postoperative bleeding [33]. Modified approaches to packing, such as using aluminum foil splints with gauze, help reduce trauma to the mucosa, thus limiting postoperative bleeding and related complications [34]. Moreover, self-adhesive wraps and elastic bandages layered over gauze have demonstrated effectiveness in maintaining necessary pressure, controlling bleeding reliably without risking distal tissue damage [35].

## ITClamp

In the context of dental hemorrhage, mechanical hemostasis devices such as hemoclips and harmonic scalpels have been used to achieve hemostasis [36]. Additionally, combining gauze packing with pressure bandaging allows for faster application times and more reliable bleeding control, especially in high-risk emergency cases [37].

Absorbable gauze, such as oxidized cellulose gauze, offers a practical alternative in cases where suturing is challenging or the bleeding source is difficult to reach. This type of gauze provides prolonged hemostasis and minimal risk of re-bleeding after surgery [38]. Elastic adhesive bandages have also shown promise in controlling hemorrhage effectively, particularly in non-compressible wounds, proving valuable for immediate application in trauma settings [32].

## Systemic Conditions and Medications Affecting Hemostasis

Systemic conditions and medications can profoundly impact hemostasis during dentoalveolar surgery, presenting unique challenges in managing bleeding risks. Conditions such as liver disease and coagulopathies disrupt natural clotting mechanisms, often necessitating specialized interventions. For instance, liver disease impairs the synthesis of clotting factors, heightening bleeding risks in oral surgeries [39]. In Crohn’s disease can increase the risk of thromboembolic events, and patients may also experience bleeding if they are on certain medications or have active disease [40]. Patients on anticoagulants, including direct oral anticoagulants (DOACs) and warfarin, pose additional risks. The anticoagulant effect complicates clotting and can lead to significant bleeding in the surgical site unless carefully managed with localized hemostatic agents [41]. In high-risk cases, tranexamic acid mouthwashes have proven effective, reducing bleeding episodes in patients without requiring systemic anticoagulant adjustments [42]. Moreover, antifibrinolytic agents like tranexamic acid help stabilize clots in anticoagulated patients, enhancing hemostasis without compromising anticoagulant therapy [43].

Medications that affect hemostasis, such as aspirin and nonsteroidal anti-inflammatory drugs (NSAIDs), further complicate surgical hemostasis. These drugs inhibit platelet function and can exacerbate bleeding risks, particularly in elderly patients who are more likely to be on multiple medications affecting coagulation [44]. Similarly, heparin-induced thrombocytopenia (HIT) presents a unique risk profile, and can sometimes be associated with bleeding, especially if the patient is on heparin and the condition is not recognized and managed promptly, the primary concern is thrombosis [45]. Topical hemostatic agents, such as HemCon Dental Dressing, have been developed to address these systemic challenges by providing immediate, localized control of bleeding. Studies show that these agents significantly reduce bleeding time and improve healing outcomes in patients on anticoagulant therapy [46]. Additional systemic hemostatic agents, such as aminocaproic acid and aprotinin, have shown mixed efficacy in surgical settings, demonstrating benefits in reducing transfusion requirements yet presenting thromboembolic risks that require careful patient selection [45].

## Preoperative Planning and Patient Preparation

Effective preoperative planning and patient preparation are vital for successful outcomes in dentoalveolar surgery. Preoperative assessments, which include evaluating the patient’s physical and psychological readiness, can significantly reduce postoperative complications. Studies on "prehabilitation" have shown that optimizing patients’ nutrition, physical condition, and mental preparedness before surgery enhances recovery and shortens hospital stays [47]. Comprehensive preoperative education also empowers patients and their families, reducing anxiety and improving postoperative compliance, ultimately leading to better outcomes [48].

High-risk patients, such as those with advanced liver disease or those requiring anticoagulation, benefit from specialized preoperative planning that includes tailored risk assessments and collaboration with multidisciplinary teams. For example, virtual surgical planning has been shown to improve accuracy and safety in complex cases, particularly in implant surgeries, by allowing for precise preoperative visualization [49]. Furthermore, studies highlight the importance of advance care planning (ACP) for high-risk patients, as these discussions improve postoperative outcomes and support patient-centered decision-making [50]. Overall, a well-coordinated preoperative plan that includes physical, psychological, and procedural preparation is essential for enhancing surgical outcomes in diverse patient populations.

## Pharmacological Agents for Hemostasis

Topical Hemostatic Agents: Gelatin Sponges, Oxidized Cellulose, and Thrombin

Recent advancements underscore the efficacy of topical hemostatic agents such as gelatin sponges, oxidized cellulose, and thrombin for achieving reliable hemostasis across various surgical settings. Gelatin sponges combined with thrombin, for instance, have demonstrated impressive outcomes in reducing time to hemostasis. A study in a porcine liver model highlighted that this combination, especially with recombinant thrombin, controlled bleeding significantly faster than oxidized cellulose alone, which is particularly beneficial in cases with high initial bleeding rates [51]. Similarly, oxidized cellulose sponges have been developed to include antimicrobial properties, offering both bleeding control and infection prevention. These sponges are bioabsorbable and were shown to manage bleeding effectively in a murine liver model while also minimizing infection risk, making them ideal for trauma settings [52].

The development of TEMPO-oxidized cellulose nanofibers combined with gelatin and thrombin has introduced a novel biocomposite sponge that further enhances hemostasis. This innovative sponge improves blood coagulation and reduces blood loss in liver hemorrhage models, offering a cost-effective and efficient solution for rapid bleeding control [53]. Gelatin-thrombin matrix sealants, commonly used in neurosurgery, provide effective hemostasis for delicate tissues while avoiding excessive inflammation or compression on surrounding tissues. This method has been particularly well-documented in both cranial and spinal surgeries, underscoring its versatility and safety in complex surgical environments [54].

Chitosan-gelatin-oxidized cellulose sponges represent another advancement, combining bioabsorbable materials to enhance blood absorption and clotting ability. These sponges have demonstrated significant efficacy in hepatic trauma models, highlighting their value for surgical bleeding control in high-risk environments [55]. Additionally, a two-layer gelatin sheet has shown superior hemostatic control in splenic models when compared to traditional hemostats. This material rapidly absorbs blood and activates clotting components directly at the bleeding site while minimizing inflammation, proving beneficial in surgeries where inflammation control is crucial [56].

Fibrocaps, a dry-powder fibrin sealant containing thrombin and fibrinogen, has shown high efficacy in reducing time to hemostasis across vascular and hepatic procedures, particularly for patients on anticoagulant therapy. This sealant offers a versatile option for controlling bleeding in a variety of surgical contexts [57]. In neurosurgical settings, customized GelFoam wafers have been highly effective for achieving hemostasis in brain surgeries. Easily prepared and adapted for specific surgical needs, these wafers provide reliable bleeding control with minimal complications, making them a practical choice for neurosurgeons [58].

## Antifibrinolytic Agents (e.g., Tranexamic Acid) in Dentoalveolar Surgery

Recent studies demonstrate that tranexamic acid (TXA) is an effective antifibrinolytic agent for bleeding control in dentoalveolar surgeries, especially among patients on anticoagulants. The EXTRACT-NOAC trial explored the use of a 10% TXA mouthwash for patients on non-vitamin K oral anticoagulants undergoing dental extractions. Results indicated that while TXA did not significantly reduce immediate post-extraction bleeding compared to placebo, it was associated with fewer delayed bleeding events and reduced unplanned medical contacts, underscoring its value in managing postoperative complications for patients on anticoagulants [59]. In a systematic review and meta-analysis focusing on anticoagulated patients, local TXA application effectively minimized postoperative bleeding risk in minor oral surgeries. This approach compared favorably against other hemostatic agents and showed no increase in thromboembolic events, highlighting TXA’s safety and efficacy when applied topically [60].

Further supporting TXA’s application in dental settings, a study found that gauze soaked in 4.8% TXA significantly reduced bleeding times in anticoagulated patients following extractions. This intervention was particularly effective in reducing immediate bleeding in surgery room or office, proving beneficial in patients who cannot safely pause anticoagulation therapy [61]. Another study confirmed these results, showing that topical TXA provides reliable hemostasis without impacting the continuation of anticoagulant medication in complex cases [62]. The mounting evidence for TXA’s safety and efficacy in managing intraoperative and postoperative bleeding in dental procedures has strengthened its role as a reliable hemostatic agent.

## Overview of Local and Systemic Hemostatic Drugs

Local and systemic hemostatic agents play a crucial role in managing bleeding during dental and oral surgeries. Local agents, such as gelatin sponges, oxidized cellulose, and chitosan-based dressings, provide effective hemostasis by forming barriers at the bleeding site and enhancing clot stability. For instance, gelatin-thrombin combinations and chitosan-derived dressings have shown high efficacy in reducing bleeding time and promoting wound healing in dental patients, even those on anticoagulation therapy [63]. Oxidized cellulose materials, commonly used in various surgeries, are effective due to their absorbable nature and antibacterial properties, making them particularly valuable in contaminated surgical fields [52]. Systemically, antifibrinolytic drugs such as tranexamic acid are used to control bleeding in patients undergoing high-risk surgeries or those with bleeding disorders by stabilizing clots and minimizing blood loss [64]. Both local and systemic agents provide tailored hemostatic support, allowing for safe and effective management of bleeding across diverse surgical contexts.

## Advanced Hemostatic Techniques

Hemostatic Products: Fibrin Sealants, Collagen-based Agents

Advanced hemostatic techniques using fibrin sealants and collagen-based agents have become essential tools in modern surgery, providing efficient bleeding control and promoting tissue repair in complex procedures. Fibrin sealants, such as TachoSil, have been highly effective in surgeries involving high-risk bleeding areas, like the liver and cranial regions. In a multicenter trial on liver resection patients, TachoSil achieved hemostasis significantly faster than oxidized regenerated cellulose, reducing both intraoperative and postoperative blood loss [65]. Another study in cranial surgery demonstrated TachoSil’s efficacy in managing bleeding from cerebral venous sinuses, a challenging area due to the risk of venous sinus tears. The study reported that TachoSil, applied as a tissue-glue-coated collagen sponge, achieved hemostasis within four minutes, highlighting its utility in neurosurgical settings [66].

Collagen-based hemostatic agents also offer promising results, especially in applications requiring absorbable materials that facilitate wound healing. Recent innovations, such as the bio-inspired V-3D-Ag-col, a "cotton-like" collagen-chitin biomaterial, have shown improved platelet adhesion and clot formation, proving particularly effective in trauma and wound management. This material not only controls bleeding but also promotes cellular growth, supporting tissue repair [67]. The newly developed sFilm-FS, a fibrin sealant co-polymeric film, has further demonstrated its capability in reducing blood loss in liver and spleen models, showing comparable effectiveness to leading hemostatic agents such as EVARREST while being biodegradable and safe for human application [68].

A systematic review on the efficacy of fibrin-based agents in controlling blood loss during liver surgeries highlighted that these agents, including fibrin glues and patches, were particularly effective in patients with coagulation deficiencies or extensive resection areas. They demonstrated significant reductions in postoperative complications and transfusion requirements, reinforcing their value in hepatic and other high-blood-loss surgeries [69]. Furthermore, the combination of fibrin sealants with other materials, such as polyglycolic acid, has been explored in liver and soft tissue surgeries. A randomized controlled study comparing a fibrin-collagen composite with polyglycolic acid found that this combination minimized biliary leakage and other complications, thus improving recovery outcomes in liver resections [70]. The growing body of evidence underscores the effectiveness of fibrin sealants and collagen-based agents in managing surgical bleeding, enhancing hemostasis, and supporting tissue healing. These agents provide tailored hemostatic solutions for various surgical challenges, improving patient outcomes by minimizing blood loss and reducing recovery times.

## Emerging Materials and Technologies for Bleeding Control

**Table:** Table1. Summry of Methods of Bleeding CFontrol

**Category**	**Method**	**Description**	**Key Benefits**	**Common Uses**
**Conventional Techniques**	Gauze Packing	Application of multiple layers of gauze with manual pressure.	Effective for primary hemorrhage control, easy to apply.	Dentoalveolar surgeries, minor bleeding.
	Sutures	Securing clots and stabilizing tissues by closing small vessels.	Useful for managing postoperative bleeding, provides long-term hemostasis.	Closing surgical incisions, managing small vessel bleeding.
	Pressure Application	Direct manual pressure with gauze or elastic bandages.	Quick and effective, can be used in emergency settings.	Immediate hemorrhage control, trauma settings.
	Enhanced Gauze (e.g., HemCon)	Hemostatic-embedded gauze to reduce bleeding time.	Faster hemostasis, improved healing in anticoagulated patients.	Dentoalveolar surgeries, patients on anticoagulants.
	Elastic Adhesive Dressings	Applied over gauze to maintain compression.	Reliable compression without compromising blood flow.	Challenging areas, non-compressible wounds.
**Pharmacological Agents**	Gelatin Sponges	Absorbable sponges that promote clot formation.	Effective for moderate bleeding, absorbable.	Various surgical settings, including dental and trauma.
	Oxidized Cellulose	Absorbable material that forms a physical barrier and promotes clotting.	Antibacterial properties, effective in contaminated fields.	Trauma, surgical wounds.
	Thrombin	Enzyme that converts fibrinogen to fibrin, promoting clot formation.	Rapid hemostasis, effective in high-bleeding scenarios.	Liver surgeries, cranial surgeries.
	Tranexamic Acid	Antifibrinolytic agent that stabilizes clots.	Reduces postoperative bleeding, safe for anticoagulated patients.	Dentoalveolar surgeries, minor oral procedures.
**Advanced Hemostatic Techniques**	Fibrin Sealants	Biologic adhesives that promote clot formation and tissue adhesion.	Effective in high-risk bleeding areas, reduces blood loss.	Liver resections, cranial surgeries.
	Collagen-Based Agents	Absorbable materials that promote clot formation and tissue repair.	Biodegradable, promotes wound healing.	Liver and soft tissue surgeries.
	Chitosan-Based Dressings	Antibacterial, biodegradable materials that enhance clot formation.	Effective in trauma and wound management, promotes healing.	Trauma, surgical wounds.
**Laser-Assisted Methods**	Diode Lasers	Use of laser energy to coagulate small vessels and achieve hemostasis.	Precise, reduces tissue damage, effective in anticoagulated patients.	Oral surgeries, tonsillectomy.
	Erbium Lasers	Laser energy that coagulates tissue and promotes hemostasis.	Effective in patients with compromised clotting abilities, reduces bleeding.	Tooth extractions, intraoral excisions.
	CO2 Lasers	Laser energy that minimizes intraoperative bleeding and improves visualization.	Reduces bleeding, allows for clearer visualization and faster intervention.	Biopsies of oral mucosal lesions, intraoral surgeries.

Emerging materials and technologies for bleeding control have introduced highly effective solutions in surgical and trauma settings. For instance, engineered nanoclay composites like kaolinite-based nanostructures are gaining traction for their rapid activation of coagulation cascades, achieving hemostasis effectively in both battlefield and civilian trauma scenarios [71]. Similarly, chitosan-based biomaterials are being developed for their antibacterial, biodegradable, and hemostatic properties, making them valuable in high-risk bleeding contexts. These chitosan composites have demonstrated significant efficacy in wound healing, clot formation, and infection prevention in surgical and trauma applications [72].

Advanced bioadhesive technologies, such as short peptide nanofiber biomaterials, offer new ways to control bleeding on complex surfaces like gastrointestinal and mucosal wounds. These nanofibers provide robust and localized hemostasis, enabling safer and faster bleeding control even in challenging areas [73]. Additionally, epinephrine-entrapped chitosan nanoparticles layered with gelatin nanofibers have shown promising results in controlling hemorrhage by enhancing platelet aggregation, making them effective in both minor and extensive bleeding cases [74]. Hydrogel-based materials also represent a significant advancement, especially in trauma care. These hydrogels, composed of natural polysaccharides and proteins, offer biocompatibility and tunable mechanical properties that aid in rapid clot formation, wound healing, and infection control. Studies confirm that these hydrogels can be injected directly into bleeding sites, forming strong and flexible barriers that promote coagulation and accelerate recovery [75]. These emerging biomaterials reflect a new generation of hemostatic agents that enhance control of bleeding and offer additional wound-healing benefits, significantly advancing bleeding management in both surgical and emergency care contexts.

## Laser-assisted Bleeding Control Methods

Laser-assisted methods have demonstrated substantial efficacy in bleeding control during various surgical procedures, especially for patients with compromised hemostasis. For instance, diode lasers have shown promise in oral surgeries, particularly in managing patients on anticoagulants. A study on anticoagulated rats undergoing oral soft tissue surgery reported complete hemostasis when a diode laser was used, offering an alternative to modifying anticoagulation therapy prior to surgery [22]. Another study exploring intraoperative bleeding control found that a 970 nm diode laser at optimized power settings could effectively coagulate small vessels without causing surrounding tissue damage, which is particularly valuable in tonsillectomy and other delicate procedures [76]. For dental implant surgeries, high-power diode lasers have also proven beneficial, reducing surgical time and minimizing bleeding compared to conventional scalpel methods. This reduction in bleeding during and after surgery helps improve patient comfort and decrease the need for postoperative interventions [77]. In patients with thrombocytopenia undergoing tooth extractions, the use of erbium lasers was associated with reduced bleeding times and faster healing, suggesting that erbium lasers are well-suited for patients with compromised clotting abilities [78].

Lasers also offer advantages in intraoral excisions, providing better control of intraoperative bleeding and reducing the need for electrocautery. In a randomized trial, CO2 lasers were particularly effective in minimizing bleeding during biopsies of oral mucosal lesions, allowing for clearer visualization and faster intervention [79]. Overall, laser-assisted bleeding control methods continue to evolve, offering precision, reduced intraoperative bleeding, and enhanced healing, making them essential tools for managing bleeding in both routine and high-risk surgical procedures.

## Conclusion

In conclusion, advancements in local bleeding control are transforming surgical and dental care, providing clinicians with more precise and effective tools to manage blood loss. Novel local bleeding control products, such as synthetic biomaterials like PTFE strips and resorbable agents like PuraStat®, are proving essential in high-risk surgeries by promoting rapid hemostasis without adverse effects. Table-1 summarised methods of bleeding control in dental procedures. Personalized approaches, enhanced by genetic profiling and point-of-care viscoelastic assays, further support tailored local bleeding control based on individual coagulation profiles, reducing reliance on generalized treatments and improving patient outcomes. In oral surgery, the use of agents like calcium sulfate and targeted applications of tranexamic acid demonstrates the ongoing effectiveness of local bleeding control, especially for anticoagulated patients. By achieving safe, localized hemostasis, these methods prevent excessive blood loss and reduce postoperative complications. The future of bleeding management lies in refining local bleeding control techniques to cater to specific surgical and patient needs, especially for populations with unique bleeding challenges, such as those with coagulopathies, pediatric, and geriatric patients. Continued research will further establish best practices, ensuring that local bleeding control methods deliver optimal safety, efficacy, and patient satisfaction across diverse medical and dental applications.

## Conflict of Interest

The authors have no competing interests to declare that are relevant to the content of this article.
